# Supplementary material for: Body Image and the Double Burden of Nutrition among South Africans from Diverse Sociodemographic Backgrounds: SANHANES-1
Source: Int J Environ Res Public Health. 2020 Jan 31;17(3):887. doi: 10.3390/ijerph17030887 (PMC7037858; doi:10.3390/ijerph17030887)
Supplement: Supplementary file 1 [file ijerph-17-00887-s001.pdf]

TableS1. Body image and the double burden of nutrition among South Africans from diverse sociodemographic backgrounds: SANHANES-1.

Supplement 1: Mean body mass index and BMI categories by demographic characteristics, in adults aged 15 years and older, SANHANES 2012[1]

				**Body Mass Index (BMI)**							
				**Underweight: BMI <18.5 kg/m2**		**Normal Weight: BMI 18.5-24.9 kg/m2**		**Overweight: BMI 25-29.9 kg/m2**		**Obese: BMI >=30 kg/m2**	
		**Mean BMI**	**95% CI**	**%**	**95% CI**	**%**	**95% CI**	**%**	**95% CI**	**%**	**95% CI**
**Sex**											
Males	2246 (42.4)	23,88(-0,22)	[23.44-24.32]	11,6	[9.7-13.9]	55,9	[52.9-58.8]	20,5	[18.1-23.2]	12,0	[9.9-14.4
Females	4165 (57.6)	28,96(-0,21)	[28.55-29.37]	3,7	[3.0-4.6]	32,0	[29.9-34.1]	24,9	[23.0-26.9]	39,4	[37.0-41.9]
**Age**											
15 to 18	764(12.4)	21,86(-0,22)	[21.43-22.30]	14,0	[10.7-18.0]	65,1	[60.4-69.6]	15,7	[12.4-19.6]	5,2	[3.5-7.7]
19 to 24	985(16.5)	24,50(-0,29)	[23.93-25.07]	9,2	[6.0-13.9]	57,0	[52.3-61.6]	17,9	[14.9-21.2]	15,9	[13.2-19.1]
25 to 34	1082(15.7)	26,83(-0,29)	[26.27-27.39]	4,2	[3.0-5.7]	44,8	[40.8-48.9]	24,9	[21.3-28.8]	26,1	[22.6-30.0]
35 to 44	980(14.5)	27,82(-0,35)	[27.14-28.51]	6,3	[4.7-8.3]	38,1	[33.7-42.7]	22,1	[18.4-26.2]	33,6	[29.3-38.1]
45 to 54	1022(15.8)	29,16(-0,42)	[28.33-29.98]	5,1	[3.5-7.4]	29,6	[25.4-34.0]	26,0	[21.2-31.4]	39,3	[34.3-44.6]
55 to 64	856(13.5)	28,93(-0,50)	[27.96-29.91]	6,6	[4.6-9.2]	27,4	[22.7-32.7]	26,4	[22.6-30.5]	39,6	[34.5-45.0]
65 and above	722(11.5)	28,44(-0,42)	[27.62-29.26]	4,6	[3.3-6.4]	31,6	[27.3-36.3]	29,2	[24.9-33.8]	34,7	[30.0-39.6]
**Race**											
African	4528(82.4)	26,83(-0,19)	[26.46-27.20]	6,9	[5.8-8.3]	42,5	[40.5-44.4]	22,7	[20.9-24.6]	27,9	[26.0-29.8]

White	135(3.3)	28,72(-1,00)	[26.75-30.69]	0,6	[0.1-3.0]	31,8	[20.0-46.5]	27,4	[17.9-39.5]	40,2	[27.4-54.5]	
Coloured	1428(11.8)	26,44(-0,32)	[25.81-27.07]	7,0	[5.4-9.0]	43,3	[39.0-47.8]	24,0	[21.0-27.3]	25,7	[21.9-29.8]	
Indian	299(2.6)	25,21(-1,42)	[22.42-28.00]	20,0	[8.4-40.7]	35,6	[28.7-43.2]	22,6	[13.2-35.9]	21,8	[12.0-36.1]	
**Locality**												
Urban formal	3084(51.1)	27,46(-0,28)	[26.91-28.01]	5,8	[4.3-7.8]	39,2	[36.6-42.0]	24,1	[21.6-26.9]	30,9	[27.9-34.0]	
Urban informal	788(9.9)	26,39(-0,41)	[25.58-27.21]	9,5	[5.7-15.4]	42,5	[37.2-48.0]	23,9	[20.0-28.2]	24,1	[20.6-28.0]	
Rural informal	1621(30.5)	26,26(-0,23)	[25.80-26.72]	7,5	[6.0-9.4]	45,0	[42.4-47.7]	21,2	[19.2-23.4]	26,2	[24.0-28.5]	
Rural formal	918(8.5)	25,32(-0,32)	[24.69-25.94]	10,3	[8.2-12.8]	48,2	[43.5-52.9]	21,9	[16.8-28.2]	19,6	[16.4-23.2]	
**Province**												
Western Cape	1052(12.3)	27,03(-0,37)	[26.31-27.76]	5,2	[3.8-7.1]	40,0	[35.5-44.8]	26,3	[22.6-30.4]	28,4	[23.8-33.5]	
Eastern Cape	802(11.6)	26,39(-0,34)	[25.72-27.05]	7,4	[5.5-9.9]	44,0	[39.8-48.4]	20,0	[17.0-23.3]	28,6	[25.1-32.4]	
Northern Cape	399(2.4)	26,27(-0,78)	[24.73-27.80]	9,4	[5.7-15.0]	45,0	[36.9-53.5]	20,2	[16.7-24.2]	25,5	[19.8-32.2]	
Free State	468(5.7)	26,89(-0,49)	[25.93-27.85]	7,8	[5.7-10.7]	42,3	[35.0-49.9]	22,0	[18.3-26.2]	27,9	[23.5-32.7]	
Kwazulu Natal	893(17.6)	27,13(-0,46)	[26.23-28.03]	7,9	[4.5-13.5]	39,3	[34.9-43.8]	22,7	[19.5-26.1]	30,2	[26.0-34.7]	
North West	789(7.0)	24,81(-0,28)	[24.27-25.36]	13,0	[9.6-17.4]	49,1	[44.7-53.5]	16,3	[13.1-20.2]	21,5	[18.2-25.3]	
Gauteng	739(24.7)	27,71(-0,47)	[26.79-28.64]	4,8	[2.8-8.0]	39,4	[35.2-43.8]	26,2	[21.8-31.1]	29,6	[24.8-34.9]	
Mpumalanga	720(7.5)	26,63(-0,46)	[25.72-27.54]	6,0	[3.8-9.3]	45,3	[41.1-49.5]	22,6	[19.9-25.7]	26,1	[21.7-31.1]	
Limpopo	549(11.1)	25,91(-0,32)	[25.29-26.53]	8,5	[6.3-11.5]	45,6	[40.9-50.4]	21,8	[17.3-27.0]	24,0	[20.7-27.8]	

**Marital Status**										
Never married	2404(45.8)	25,26(-0,23)	[24.81-25.71]	9,1	[7.1-11.6]	50,3	[47.6-53.0]	20,1	[17.7-22.7]	20,5	[18.3-22.8]
Living together	462(8.0)	27,01(-0,45)	[26.12-27.90]	5,9	[4.0-8.5]	41,7	[35.5-48.2]	29,1	[23.5-35.4]	23,3	[18.4-29.0]
Married	1817(34.7)	28,75(-0,31)	[28.13-29.36]	3,9	[2.9-5.3]	31,4	[28.1-34.8]	27,3	[24.4-30.5]	37,4	[33.8-41.0]
Widowed/Separated/Divorced	639(11.5)	29,87(-0,53)	[28.84-30.91]	4,8	[3.0-7.4]	27,2	[22.8-32.0]	24,1	[20.1-28.6]	43,9	[38.2-49.8]
**Highest level of Education**											
No schooling	554(8.9)	26,94(-0,49)	[25.97-27.91]	12,4	[7.9-19.0]	34,0	[29.3-39.1]	23,3	[19.3-27.9]	30,2	[25.6-35.3]
Grade 0-5	645(11.1)	27,06(-0,50)	[26.08-28.03]	9,6	[6.8-13.5]	38,3	[34.0-42.9]	21,0	[16.7-26.1]	31,0	[25.9-36.6]
Grade 6-7	643(11.1)	27,10(-0,43)	[26.25-27.95]	7,7	[5.4-11.0]	38,9	[33.3-44.8]	25,8	[21.0-31.3]	27,6	[23.1-32.6]
Grade 8-11	2185(39.1)	26,38(-0,25)	[25.89-26.86]	7,3	[5.5-9.6]	45,4	[42.4-48.3]	21,3	[18.8-23.9]	26,1	[23.6-28.7]
Grade 12	1083(22.1)	26,70(-0,32)	[26.07-27.33]	4,6	[2.7-7.5]	46,8	[42.3-51.4]	23,2	[18.9-28.2]	25,4	[21.5-29.7]
Higher education	316(7.8)	28,35(-0,62)	[27.14-29.56]	1,2	[0.5-2.7]	31,4	[23.9-40.0]	31,8	[24.3-40.3]	35,6	[27.5-44.7]
**Annual Income Category[2] ZAR[1]**											
No income	1413(34.0)	26,51(-0,31)	[25.89-27.13]	8,2	[5.9-11.2]	46,6	[43.3-49.9]	18,9	[16.2-21.8]	26,3	[23.5-29.3]
1-9600	558(14.3)	27,51(-0,46)	[26.61-28.42]	7,0	[4.4-10.9]	36,6	[31.4-42.0]	22,5	[18.6-27.0]	33,9	[28.4-39.8]
9601-38400	1593(38.6)	27,77(-0,33)	[27.12-28.41]	5,9	[4.2-8.2]	37,7	[34.2-41.3]	24,7	[21.4-28.2]	31,8	[28.1-35.7]
38401-153600	320(9.8)	28,23(-0,52)	[27.21-29.24]	1,2	[0.5-2.8]	30,6	[22.6-40.1]	32,3	[23.4-42.7]	35,8	[28.0-44.5]

[1] 1 USD to ZAR = 14.63 ZAR

>=153601	97(3.2)	29,13(-0,84)	[27.48-30.79]	0,2	[0.0-1.8]	25,0	[13.9-40.9]	33,5	[18.0-53.5]	41,3	[24.5-60.3]
**Total**	6411(100)	26,81(-0,17)	[26.47-27.14]	7,0	[6.0-8.3]	42,1	[40.4-43.9]	23,0	[21.4-24.7]	27,8	[26.1-29.6]

aWeighted percentage to represent the South African ethnic diversity, geographic location (in terms of provinces; urban/rural) and gender, based on the 2011

Census [25]

bBMI percentiles used to categorise weight status in children 15-18 years

cFor persons 18 years and older

dSEM – Standard error of the mean

1. Of the individuals for whom valid weight and height readings were recorded, and who answered the questions on perceived and ideal body silhouettes

2. For persons aged 18 years and older

Table S2. The likelihood of body image discrepancy (BID) and Feel-Ideal Difference (FID) among participants from the SANHANES-1 survey by indicators of sociodemographic factors (marital status-adjusted odds ratios (ORs) from multinomial regression).

	Adjusted for Marital Status					
	Body image discrepancy (BID)			Feel-Ideal Difference (FID)		
**Socio-demographic factors**	**less than zero (< 0)**	**equal to zero (= 0)**	**greater than zero (>0)**	**less than zero (< 0)**	**equal to zero (= 0)**	**greater than zero (>0)**
Gender						
Male	3.67 (2.97 - 4.54)	1 (< 0.001)	0.586 (0.473 - 0.725)	1.538 (1.292 - 1.831)	1 (< 0.001)	0.571 (0.471 - 0.693)
Female	1		1	1		1
Education level						
No schooling	1.96 (1.13 - 3.40)		0.926 (0.572 - 1.497)	0.888 (0.557 - 1.416)		0.526 (0.336 - 0.822)
Grade 0-5	1.77 (1.04 - 3.01)		1.073 (0.675 - 1.707)	1.119 (0.710 - 1.765)		0.717 (0.468 - 1.099)
Grade 6-7	1.37 (0.80 - 2.36)	1 (< 0.001)	1.102 (0.691 - 1.758)	1.059 (0.665 - 1.688)	1 (0.086)	0.712 (0.462 - 1.097)
Grade 8-11	1.14 (0.70 - 1.85)		1.052 (0.700 - 1.582)	0.899 (0.591 - 1.368)		0.731 (0.502 - 1.065)
Grade 12	0.92 (0.56 - 1.51)		0.765 (0.506 - 1.155)	0.785 (0.509 - 1.210)		0.703 (0.479 - 1.032)
Higher education	1		1	1		1
Geographic location						
Urban formal	0.834 (0.613 - 1.133)		1.215 (0.902 - 1.636)	1.240 (0.955 - 1.611)		1.995 (1.505 - 2.646)
Urban informal	0.812 (0.537 - 1.229)	1 (0.186)	1.034 (0.699 - 1.531)	1.549 (1.095 - 2.190)	1 (< 0.001)	2.346 (1.616 - 3.406)
Rural formal (Tribal areas)	0.793 (0.557 - 1.130)		1.124 (0.803 - 1.574)	1.671 (1.252 - 2.228)		1.568 (1.129 - 2.177)
Rural informal (farms)	1		1	1		1
Ethnicity						
Black	1.379 (0.829 - 2.294)		2.063 (1.323 - 3.215)	1.718 (0.995 - 2.967)		0.700 (0.465 - 1.055)
White	1.122 (0.484 - 2.598)	1 (< 0.001)	1.857 (0.934 - 3.694)	0.820 (0.329 - 2.040)	1 (< 0.001)	0.993 (0.533 - 1.852)
Mixed Ancestry	1.470 (0.879 - 2.458)		1.372 (0.872 - 2.158)	1.660 (0.951 - 2.897)		1.016 (0.669 - 1.544)
Indian/Asian	1		1	1		1
Household income (per annum) ZAR[i]						
No income	5.080 (1.996- -12.932)		0.891 (0.486 - 1.632)	1.440 (0.655 - 3.169)		0.413 (0.227 - 0.750)
≤38400	4.199 (1.601 - 11.011)		0.723 (0.382 - 1.371)	1.517 (0.674 - 3.415)	1 (< 0.001)	0.431 (0.229 - 0.810)
>38400	1		1	1		

Adjusted for **marital status** in four categories (Never married, Living together, Married, Widowed/Separated/Divorced).

Table S3. The likelihood of body image discrepancy (BID) and Feel-Ideal Difference (FID) among participants from the SANHANES-1 survey by indicators of sociodemographic factors (ethnicity-adjusted odds ratios [ORs] from multinomial regression).

	Adjusted for Ethnicity					
	Body image discrepancy (BID)			Feel-Ideal Difference (FID)		
**Socio-demographic factors**	**less than zero (< 0)**	**equal to zero (= 0)**	**less than zero (< 0)**	**equal to zero (= 0)**	**less than zero (< 0)**	**equal to zero (= 0)**
Gender						
Male	3.874 (3.122 - 4.807)	1 (< 0.001)	0.569 (0.458 - 0.706)	1.567 (1.314 - 1.869)	1 (< 0.001)	0.562 (0.462 - 0.683)
Female	1		1	1		1
Education level						
No schooling	1.99 (1.15- 3.43)		0.950 (0.590 - 1.531)	0.928 (0.584 -1.474)		0.532 (0.341 - 0.829)
Grade 0-5	1.86 (1.10 - 0.16)		1.089 (0.688 - 1.723)	1.191 (0.759 - 1.869)		0.721 (0.472 - 1.101)
Grade 6-7	1.44 (0.84 - 2.47)	1 (< 0.001)	1.117 (0.703 - 1.775)	1.121 (0.707 - 1.779)	1 (0.061)	0.714 (0.465 - 1.096)
Grade 8-11	1.20 (0.74 - 1.93)		1.068 (0.713 - 1.598)	0.949 (0.626 - 1.437)		0.734 (0.506 - 1.066)
Grade 12	0.94 (0.58 - 1.54)		0.769 (0.510 - 1.160)	0.810 (0.527 - 1.245)		0.707 (0.482 - 1.038)
Higher education	1		1	1		1
Marital status						
Never married	1.399 (1.002 - 1.954)		0.850 (0.632 - 1.144)	0.983 (0.743 - 1.300)		0.636 (0.482 - 0.840)
Living with partner	1.153 (0.743 - 1.790)	1 (< 0.001)	0.817 (0.546 - 1.223)	1.017 (0.702 - 1.473)	1 (< 0.001)	0.832 (0.571 - 1.210)
Married	0.712 (0.511 - 0.994)		1.101 (0.826 - 1.468)	0.816 (0.617 - 1.079)		0.968 (0.742 - 1.264)
Widowed/Separated/Divorced	1		1	1		1
Geographic location						
Urban formal	0.790 (0.581 - 1.075)		1.204 (0.895 - 1.619)	1.219 (0.938 - 1.583)		1.992 (1.504 - 2.638)
Urban informal	0.763 (0.505 - 1.153	1 (0.108)	1.021 (0.692 - 1.509)	1.525 (1.080 - 2.155)	1 (< 0.001)	2.321 (1.604 - 3.360)
Rural formal (Tribal areas)	0.760 (0.534 - 1.083		1.091 (0.781 - 1.526)	1.666 (1.248 - 2.224)		1.551 (1.119 - 2.148)
Rural informal (farms)	1		1	1		1
Household income (per annum) ZAR[i]						
No income	4.836 (1.908 - 12.256)		0.907 (0.496 - 1.658)	1.495 (0.685 - 3.264)		0.415 (0.229 - 0.754)
≤38400	3.981 (1.526 - 10.390)		0.738 (0.390 - 1.395)	1.580 (0.708 - 3.528)	1 (< 0.001)	0.434 (0.231 - 0.815)
>38401	1		1	1		1

Adjusted for **ethnicity** in four categories (Black, White, Mixed Ancestry, Indian/Asian)

Table S4. The likelihood of body image discrepancy (BID) and Feel-Ideal Difference (FID) among participants from the SANHANES-1 survey by indicators of sociodemographic factors (gender-adjusted odds ratios [ORs] from multinomial regression).

	Adjusted for Gender					
	Body image discrepancy (BID)			Feel-Ideal Difference (FID)		
**Socio-demographic factors**	**equal to zero (= 0)**	**less than zero (< 0)**	**equal to zero (= 0)**	**less than zero (< 0)**	**equal to zero (= 0)**	**less than zero (< 0)**
Education level						
No schooling	1.90 (1.09 -3.28)		0.946 (0.584 - 1.530)	0.871 (0.546 - 1.391)		0.534 (0.341 - 0.837)
Grade 0-5	1.76 (1.03 - 3.01)		1.081 (0.680 - 1.720)	1.115 (0.707 - 1.759)		0.723 (0.471 - 1.108)
Grade 6-7	1.37 (0.79 -2.36)	1 (< 0.001)	1.109 (0.695 - 1.769)	1.055 (0.662 - 1.681)	1 (< 0.106)	0.715 (0.464 - 1.101)
Grade 8-11	1.14 (0.70 - 1.84)		1.058 (0.704 - 1.590)	0.897 (0.589 - 1.365)		0.732 (0.503 - 1.067)
Grade 12	0.91 (0.56 - 1.49)		0.765 (0.507 - 1.156)	0.782 (0.507 - 1.206)		0.708 (0.482 - 1.040)
Higher education	1		1	1		1
Marital status						
Never married	1.373 (0.982 - 1.920)		0.848 (0.630 - 1.143)	0.958 (0.724 - 1.269)		0.637 (0.482 - 0.841)
Living with partner	1.137 (0.732 - 1.766)	1 (< 0.001)	0.816 (0.545 - 1.222)	0.996 (0.687 - 1.444)	1 (< 0.001)	0.833 (0.572 - 1.212)
Married	0.711 (0.509 - 0.992)		1.100 (0.826 - 1.467)	0.815 (0.616 - 1.077)		0.967 (0.741 - 1.262)
Widowed/Separated/Divorced	1			1		1
Geographic location						
Urban formal	0.807 (0.592 - 1.101)		1.223 (0.906 - 1.651)	1.229 (0.943 - 1.601)		2.041 (1.536 - 2.713)
Urban informal	0.781 (0.515 - 1.185)	1 (0.131)	1.041 (0.702 - 1.544)	1.531 (1.081 - 2.168)	1 (< 0.001)	2.395 (1.647 - 3.482)
Rural formal (Tribal areas)	0.782 (0.546 - 1.119)		1.116 (0.794 - 1.568)	1.676 (1.251 - 2.247)		1.605 (1.152 - 2.238)
Rural informal (farms)	1		1	1		1
Ethnicity						
Black	1.306 (0.781 - 2.182)		2.159 (1.380 - 3.377)	1.664 (0.962 - 2.878)		0.711 (0.471 - 1.075)
White,	1.091 (0.468 - 2.543)	1 (< 0.001)	1.880 (0.944 - 3.742)	0.813 (0.326 - 2.024)	1 (< 0.001)	0.997 (0.535 -1.860)
Mixed Ancestry	1.433 (0.854 - 2.406)		1.407 (0.894 - 2.216)	1.633 (0.935 - 2.852)		1.028 (0.676 - 1.563)
Indian/Asian	1		1	1		1
Income category(per annum) ZAR[i]						
No income	4.602 (1.802 - 11.748)		0.914 (0.498 - 1.678)	1.380 (0.626 - 3.040)		0.420 (0.231 - 0.764)
≤38400	3.788 (1.440 - 9.964)		0.746 (0.393 - 1.416)	1.451 (0.644 - 3.271)		0.440 (0.234 - 0.829)
>38400	1		1	1		1

Adjusted for **gender** in two categories (Men and Women)

Table S5. The likelihood of body image discrepancy (BID) and Feel-Ideal Difference (FID) among participants from the SANHANES-1 survey by indicators of sociodemographic factors (income-adjusted odds ratios [ORs] from multinomial regression).

			**Adjusted for income**			
		**Body image discrepancy (BID)**			**Feel-Ideal Difference (FID)**	
**Socio-demographic factors**	**equal to zero (= 0)**	**less than zero (< 0)**	**equal to zero (= 0)**	**less than zero (< 0)**	**equal to zero (= 0)**	**less than zero (< 0)**
Gender						
Male	3.922 (3.165 - 4.860)	1 (< 0.001)	0.588 (0.475 - 0.728)	1.540 (1.293 -1.834)	1 (< 0.001)	0.564 (0.465 - 0.684)
Female	1		1	1		1
Education level						
No schooling	2.120 (1.254 - 3.584)		0.792 (0.503 - 1.248)	0.915 (0.585 - 1.431)		0.456 (0.298 - 0.696)
Grade 0-5	1.961 (1.177 - 3.268)		0.912 (0.588 - 1.416)	1.177 (0.761 - 1.820)		0.624 (0.416 - 0.935)
Grade 6-7	1.528 (0.908 - 2.573)	1 (< 0.001)	0.937 (0.602 - 1.458)	1.113 (0.712 - 1.740)	1 (< 0.006)	0.616 (0.409 - 0.928)
Grade 8-11	1.255 (0.789 - 1.996)		0.920 (0.625 - 1.354)	0.931 (0.621 - 1.395)		0.642 (0.449 - 0.918)
Grade 12	0.993 (0.615 - 1.604)		0.695 (0.466 -1.037)	0.795 (0.521 - 1.214)		0.635 (0.437 - 0.922)
Higher education	1		1	1		1
Marital status						
Never married	1.344 (0.963 - 1.878)		0.870 (0.647 - 1.171)	0.954 (0.721 - 1.262)		0.654 (0.496 - 0.862)
Living with partner	1.112 (0.717 - 1.726)	1 (< 0.001)	0.837 (0.560 - 1.252)	0.989 (0.683 - 1.433)	1 (< 0.001)	0.855 (0.588 - 1.243)
Married	0.691 (0.496 - 0.962)		1.141 (0.858 - 1.518)	0.807 (0.611 - 1.065)		1.002 (0.769 - 1.305)
Widowed/Separated/Divorced	1		1	1		1
Geographic location						
Urban formal	0.802 (0.589 - 1.092)		1.264 (0.938 - 1.704)	1.198 (0.921 - 1.558)		2.059 (1.552 - 2.732)
Urban informal	0.776 (0.512 - 1.177)	1 (0.063)	1.068 (0.721 - 1.582)	1.507 (1.065 - 2.134)	1 (< 0.001)	2.420 (1.666 - 3.516)
Rural formal (Tribal areas)	0.775 (0.541 - 1.108)		1.140 (0.812 - 1.601)	1.655 (1.236 - 2.216)		1.625 (1.167 - 2.263)
Rural informal (farms)	1		1	1		1
Ethnicity						
Black	1.316 (0.789 - 2.196)		2.121 (1.358 - 3.315)	1.677 (0.971 - 2.897)		0.706 (0.468 - 1.065)
White,	0.987 (0.427 - 2.281)	1 (< 0.001)	1.976 (0.994 - 3.926)	0.802 (0.323 - 1.992)	1 (< 0.001)	1.055 (0.567 -1.961)
Mixed Ancestry	1.460 (0.871 - 2.445)		1.404 (0.892 - 2.209)	1.624 (0.930 - 2.834)		1.017 (0.670 - 1.543)
Indian/Asian	1		1	1		1

Adjusted for **income** in five categories (No income, ≤38400 ZAR and >38401)
